# Supplementary material for: Isolation, Characterization and Anticancer Activity of Two Bioactive Compounds from Arisaema flavum (Forssk.) Schott
Source: Molecules. 2022 Nov 16;27(22):7932. doi: 10.3390/molecules27227932 (PMC9697112; doi:10.3390/molecules27227932)
Supplement: Supplementary file 1 [file molecules-27-07932-s001.zip › molecules-1966223-supplementary.pdf]

Supplementary Data

Isolation, Characterization and Anticancer Activity of Two Bioactive Compounds from *Arisaema flavum* (Forssk.) Schott

Sobia Nisa [1,*], Yamin Bibi [2], Saadia Masood [3], Ashraf Ali [4,*], Sadia Alam [1], Maimoona Sabir [1], Abdul Qayyum [5], Waqas Ahmed [1], Sarah Alharthi [6,7], Eman Y. Santali [8], Saif A. Alharthy [9,10], Waleed M. Bawazir [9,11] and Majed N. Almashjary [9,11,12]

[1] Department of Microbiology, The University of Haripur, Haripur 22620, Pakistan
[2] Department of Botany, PMAS-Arid Agriculture University Rawalpindi, Rawalpindi 46300, Pakistan
[3] Department of Statistics & Mathematics, PMAS-Arid Agriculture University Rawalpindi, Rawalpindi 46300, Pakistan
[4] Department of Chemistry, The University of Haripur, Haripur 22620, Pakistan
[5] Department of Agronomy, The University of Haripur, Haripur 22620, Pakistan
[6] Department of Chemistry, College of Science, Taif University, P.O. Box 11099, Taif 21944, Saudi Arabia
[7] Center of Advanced Research in Science and Technology, Taif University, P.O. Box 11099, Taif 21944, Saudi Arabia
[8] Department of Pharmaceutical Chemistry, College of Pharmacy, Taif University, P.O. Box 11099, Taif 21944, Saudi Arabia
[9] Department of Medical Laboratory Sciences, Faculty of Applied Medical Sciences, King Abdulaziz University, P.O. Box 80216, Jeddah 21589, Saudi Arabia
[10] Toxicology and Forensic Sciences Unit, King Fahd Medical Research Center, King Abdulaziz University, P.O. Box 80216, Jeddah 21589, Saudi Arabia
[11] Hematology Research Unit, King Fahd Medical Research Center, King Abdulaziz University, Jeddah, 21589, Saudi Arabia
[12] Animal House Unit, King Fahd Medical Research Center, King Abdulaziz University, Jeddah, Saudi Arabia
* Correspondence: sobia@uoh.edu.pk (S.N.); ashraf.ali@uoh.edu.pk (A.A.)

---

1. *Spectroscopic analysis of isolated compounds*

1.1 Spectral analysis of compound I

UV (CH3-COO-CH2-CH3: CHCl3) 254nm; IR(KBr) cm^{-1}: 2922.8 (CH3-CH2), 1738.88 (C=O), 1461 (CH3), 1372.7 (CH3), 1179 (C-O); 1H NMR(CD3OD) δ / ppm : 4.11 (2H,dd,*J*=7.14Hz,H), 2.28 (2H,t,*J*=7.60Hz,H), 1.59 (2H,t,*J*=7.23Hz,H), 1.27 (22H,s), 1.25 (5H,m,H), 0.88 (3H,t,*J*=6.6Hz,H16); 13C NMR (CD3OD) δ / ppm: 173.9 (C=O, C-1), 60.15 (CH_2, C-17), 34.4 (CH_2, C-2), 29.708 (CH_2, C-3), 29.70 ($[CH_2]_{10}$, C4-C13), 29.27 (CH_2, C-14), 24.99(CH_2, C15-), 22.7(CH_3, C-18), 14.25 (CH_3, C16); MS *m* / *z* : m/z 284(M^+), 199 $[(CH_2)_9\ COOC_2H_5]^+$, 157 $[(CH_2)_6\ COOC_2H_5]^+$, 101 $[(CH_2)_2\ COOC_2H_5]^+$, 88 $(H_5C_2—O(OH)---C=CH_2)^+$ (Calculated for $C_{18}H_{36}O_2$:284).

1.2 Spectral analysis of compound II

UV (CH_3-COO-CH_2-CH_3: $CHCl_3$) 254nm; IR(KBr) cm^{-1}: 2924.97 (CH_3-CH_2), 2853 (CH_3), 1736 (C=O), 1463 (CH_3), 1376 (CH_3), 1171 (C-O), 1108 (C=O), 1054 (C=O), 800 (CH_3) ; 1H NMR(CD_3OD) δ/ppm : 4.11 (2H,dd,J=7.14Hz,-CH_2), 3.66 (3H,s,O-CH_3), 2.30 (4H,dd,J=7.09Hz,2CH_2), 1.59 (5H,m,CH,2CH_2), 1.28 (26H,s,13CH_2), 1.28 (4H,s,2CH_2), 0.88 (6H,m,2CH_3).; 188.45 (C=O,C-5), 174.45 (C=O, C-1), 60.23 (CH_2, C-2), 51.3 (CH_3,C-26), 34.48 ($[CH_2]_2$,C-4,6), 34.21 (CH_2, C-18), 32.01 (CH_2,C-14), 29.77-29.44 ($[CH_2]_{10}$, C9-17), 29.35 ($[CH_2]_2$, C23,24), 29.25($[CH_2]_2$, C20-21), 24.24(CH, C19-),22.7(CH_3, C-18), 14.34(CH_3, C-22), 14.20 (CH_3, C25); MS m / z : m/z 410 (M^+), 288, 199 $[(CH_2)_9\ COOC_2H_5]^+$, 157 $[(CH_2)_6\ COOC_2H_5]^+$, 101 $[(CH_2)_2\ COOC_2H_5]^+$, 88 $(H_5C_2—O(OH)---C=CH_2)^+$ (Calculated for $C_{26}H_{50}O_3$:410).

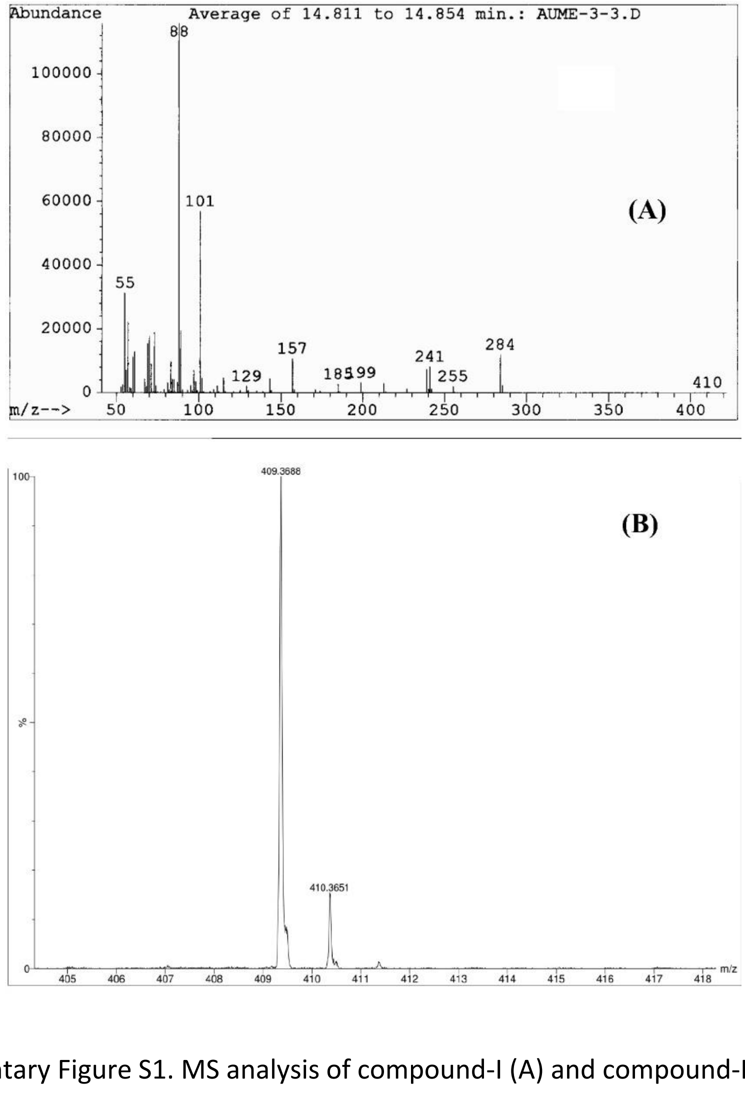

Supplementary Figure S1. MS analysis of compound-I (A) and compound-II (B)

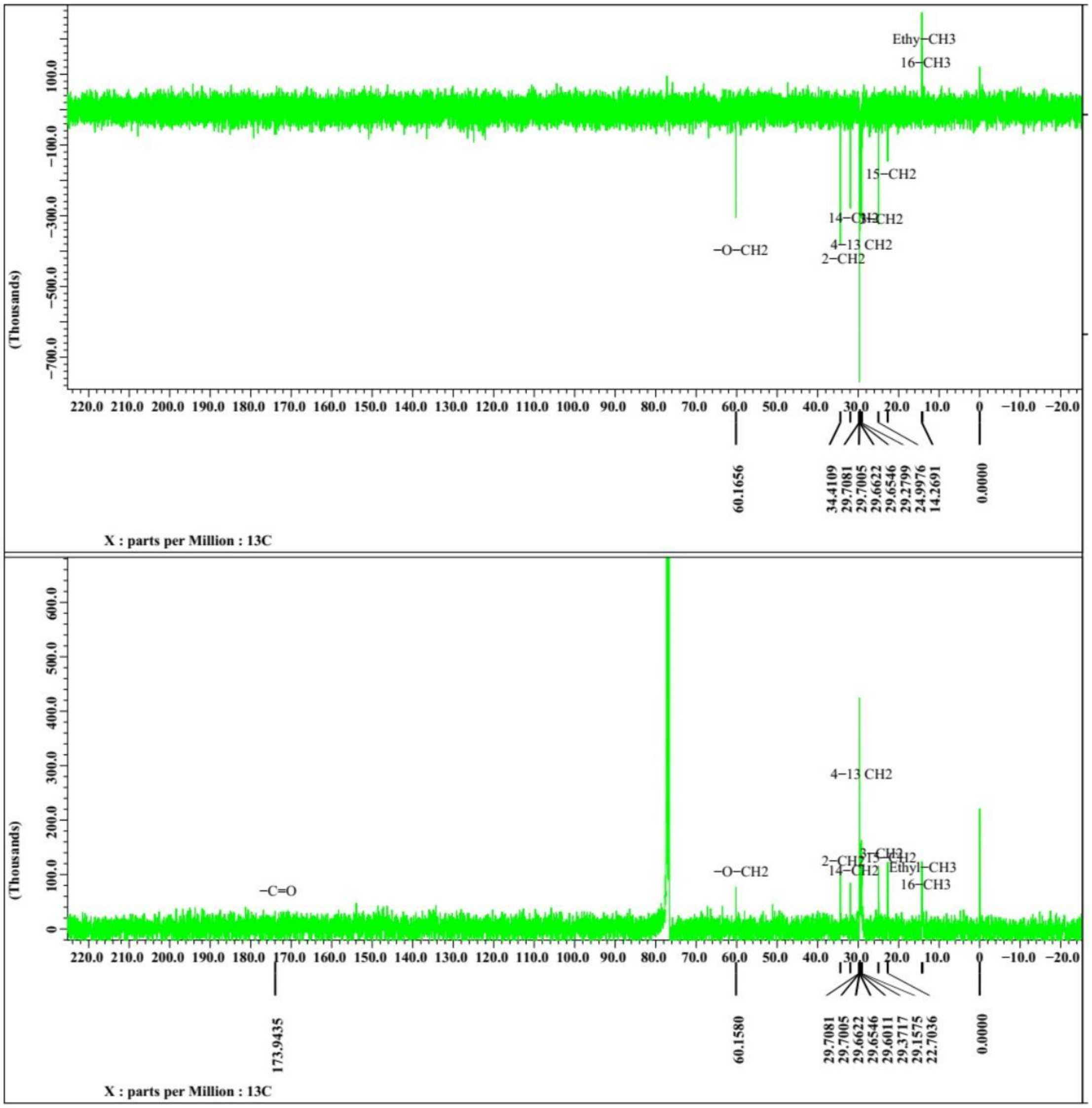

Supplementary Figure S2. Carbon DEPT and Carbon Spectrum of Hexadecanoic acid ethyl ester (upper spectrum indicate Carbon DEPT where inverted peaks are showing CH_2 and upright peaks are indicating CH and CH_3. Lower spectrum is indicating the total Carbons of Compound 1 with reference to internal standard TMS).

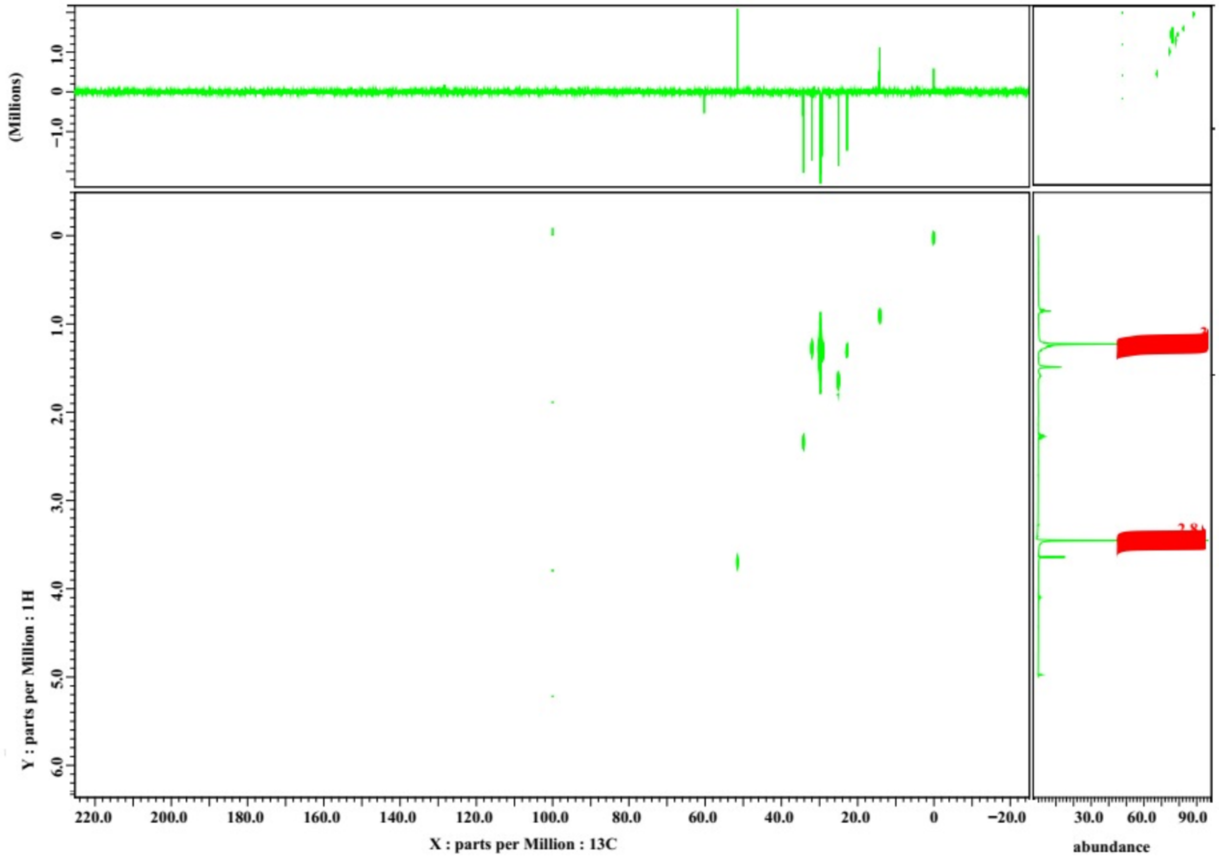

Supplementary Figure S3. HETCORE spectrum of Hexadecanoic acid ethyl ester.

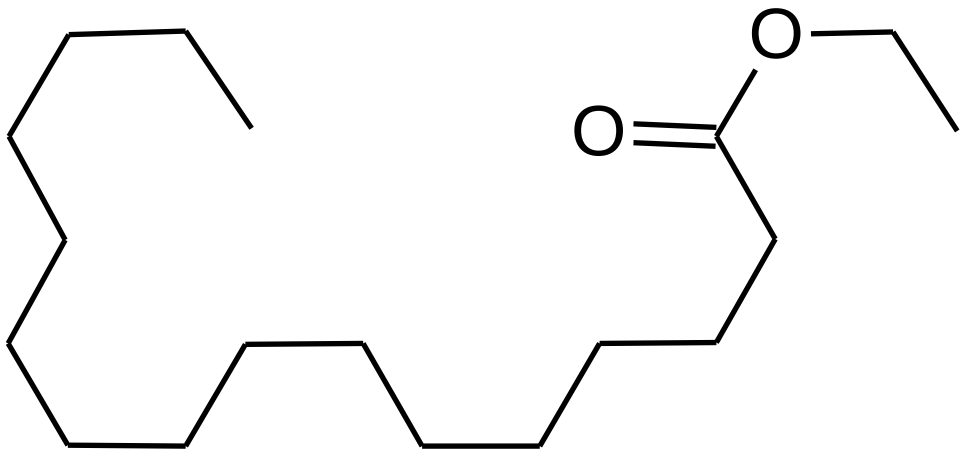

Supplementary Figure S4. Proposed structure of compound 1 identified as Hexadecanoic acid ethyl ester.

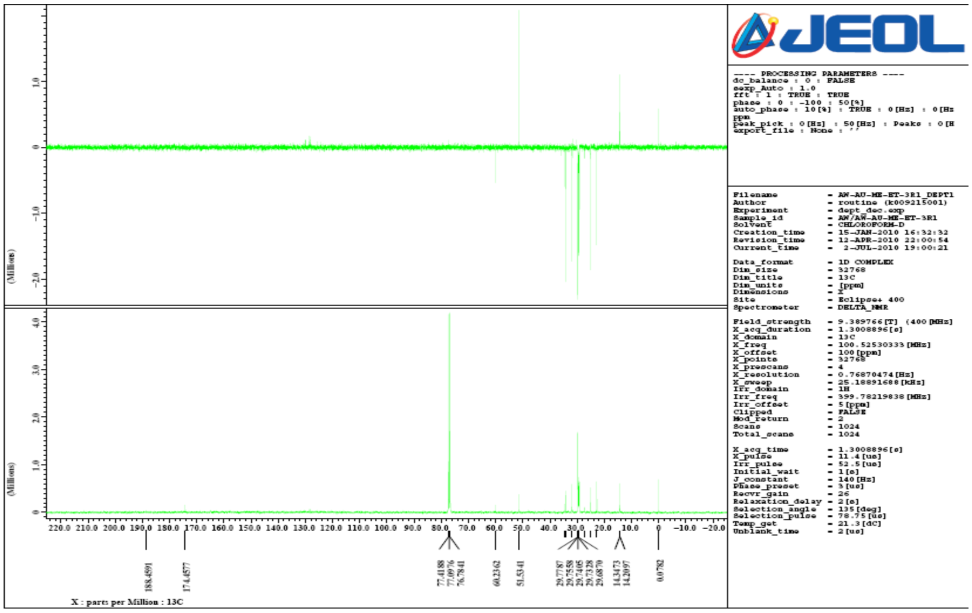

Supplementary Figure S5. Carbon DEPT and Carbon Spectrum 5-Oxo-19 propyl-docosanoic acid methyl ester (upper spectrum indicating Carbon DEPT where inverted peaks are showing CH_2 and upright peaks are indicating CH and CH_3. Lower spectrum is indicating the total Carbons of Compound 1 with reference to internal standard TMS).

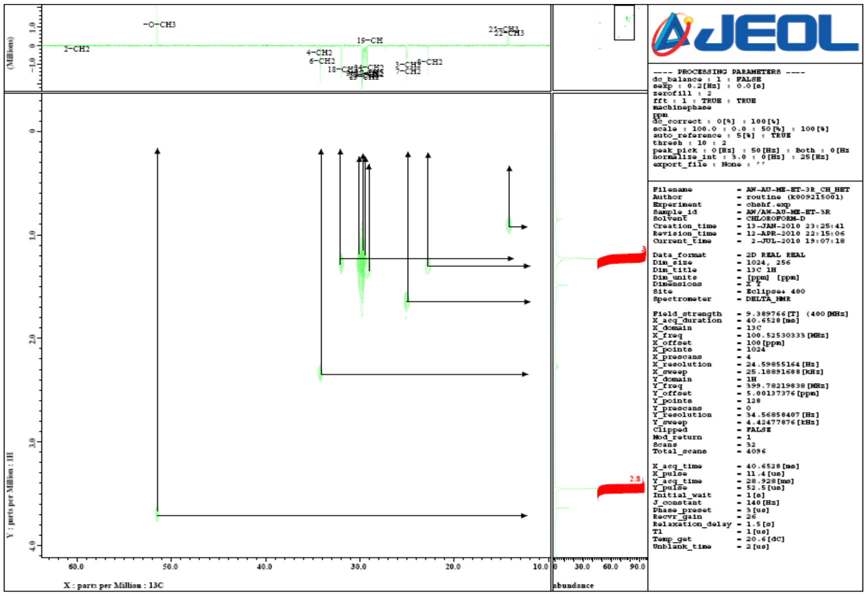

Supplementary Figure S6. HETCORE spectrum 5-Oxo-19 propyl-docosanoic acid methyl ester (Indicating Carbon spectrum on one side and Proton Spectrum on other side and their linkage in Centre).

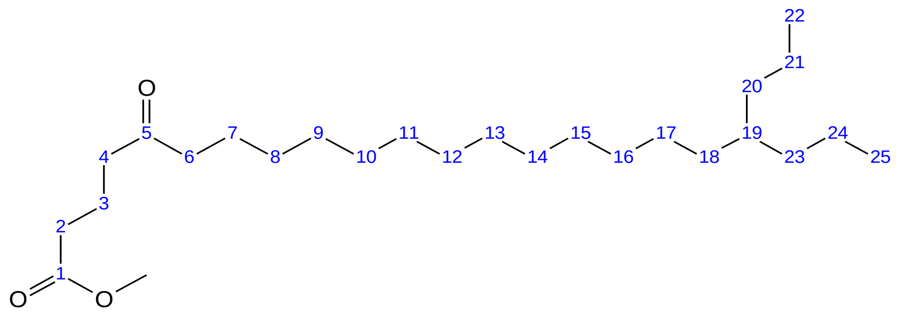

Supplementary Figure S7 Proposed structure of Compound 2 identified as 5-Oxo-19 propyl-docosanoic acid methyl ester.

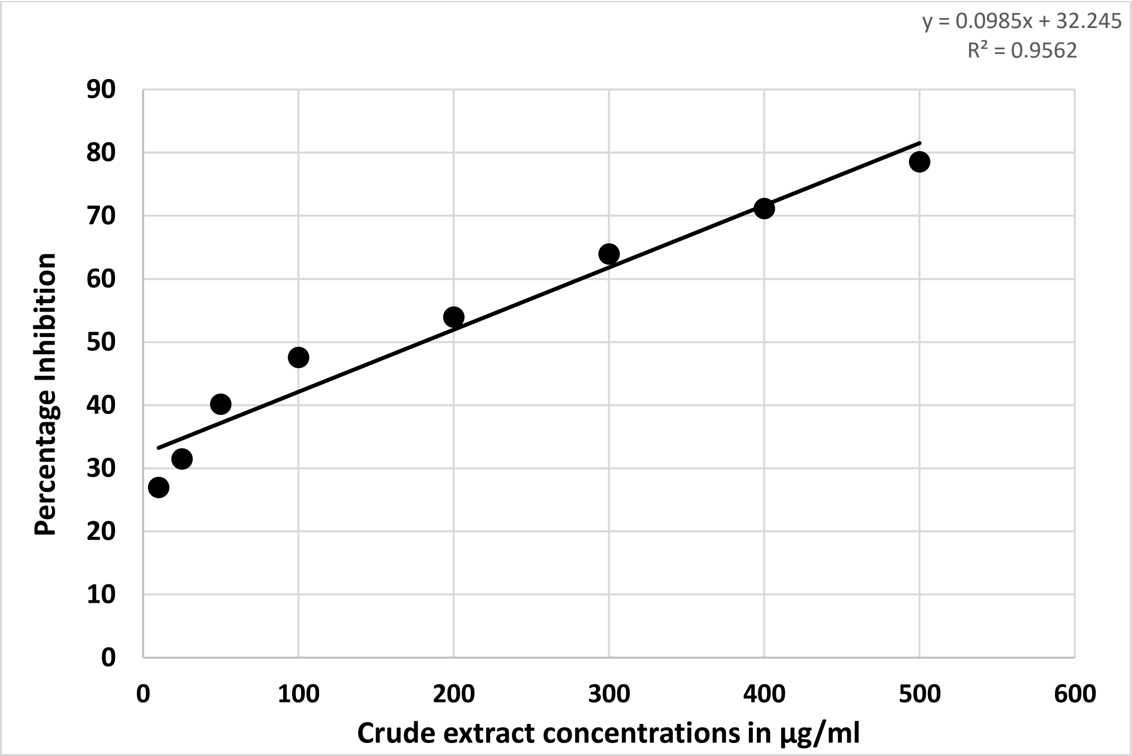

Supplementary Figure S8. Effect of concentration of *Arisaema flavum* (Forssk) crude extracts on inhibition of breast cancer cell lines (MCF 7) growth
